# High-throughput impedance monitoring in 3D tumor cultures: a multiplex, microfluidic-free platform for drug screening†

**DOI:** 10.1039/d5lc00540j

**Published:** 2025-07-17

**Authors:** Attilio Marino, Kamil Ziaja, Marie Celine Lefevre, Maria Cristina Ceccarelli, Matteo Battaglini, Carlo Filippeschi, Gianni Ciofani

**Affiliations:** a Istituto Italiano di Tecnologia, Smart Bio-Interfaces Viale Rinaldo Piaggio 34 56025 Pontedera Italy attilio.marino@iit.it kamil.ziaja@iit.it gianni.ciofani@iit.it; b Scuola Superiore Sant'Anna, The Biorobotics Institute Viale Rinaldo Piaggio 34 56025 Pontedera Italy; c University of Aveiro, Department of Chemistry, CICECO-Aveiro Institute of Materials Rua de Calouste Gulbenkian 1 3810-074 Aveiro Portugal; d Istituto Italiano di Tecnologia, Bioinspired Soft Robotics Via Morego 30 16163 Genova Italy

## Abstract

The development of an effective therapy against glioblastoma (GBM) remains a significant and unmet clinical need. To address this challenge, creating predictive, physiologically relevant screening models is essential for accelerating the identification of promising drug candidates. In this paper, we present a novel impedance-based device where two-photon polymerization-fabricated scaffolds embedding electrodes are colonized by GBM cells, effectively replicating the three-dimensional environment of the microscopic tumor foci that persist following tumor resection and cause recurrence. The results demonstrated that the proposed GBM-on-chip model enables high-throughput, multiplexed, and real-time monitoring of the development of tumor spheroids and their responses to therapeutic agents. Validation studies demonstrated the platform ability to detect subtle cytotoxic effects undetectable by traditional immunofluorescence methods, with optical transparency enabling complementary imaging analysis. This system represents a versatile framework for assessing drug efficacy in complex, physiologically relevant 3D tumor models, paving the way for innovations in cancer pharmacology.

## Abstract 2

A novel impedance-based 3D glioblastoma-on-chip platform integrates two-photon polymerization-fabricated scaffolds and multielectrode arrays for real-time, high-throughput drug screening.

## Graphical abstract

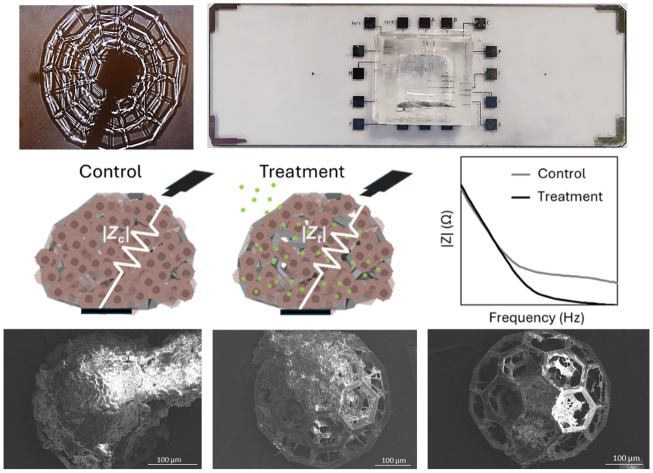

## 1. Introduction

Glioblastoma (GBM) is classified as a grade IV tumor according to the WHO classification, and is the most prevalent malignant primary brain tumor.^1,2^ Epidemiological studies indicate that GBM constitutes nearly half of all malignant tumors within the central nervous system (CNS), with patient 5-year survival rates from 3.3% to 10.1% and a median survival of less than 15 months since diagnosis. Despite advancements in diagnostic imaging, surgical intervention often fails to completely remove neoplastic tissue, and even apparently total tumor resection does not prevent GBM recurrence.^3^ This is attributed to residual GBM cells, which form microscopic foci that are undetectable and cannot be removed by standard methods. With this considered, new therapeutic strategies aim to enhance surgical tumor resection, which remains the primary treatment for GBM. These strategies involve the use of theranostic agents that facilitate the localization and elimination of GBM microscopic foci.^4,5^

Current research focuses on exploring the anticancer effects of various therapies, including oncolytic virotherapy, vaccine therapy, CAR-T therapy, monoclonal antibodies, and unconventional radiotherapy methods such as photon radiation and FLASH radiotherapy.^6^ Additionally, the investigation of small molecules remains an essential aspect of drug development.^6–8^ Nanomedicine and, in particular, theranostic approaches, which combine imaging and cancer cell death induction, offer promise in targeting residual microscopic foci. Nanoparticles aid in crossing the blood–brain barrier (BBB), enabling the delivery of anticancer agents to the brain; drug delivery systems based on nanomaterials enhance the therapeutic efficacy of existing GBM treatments, such as temozolomide (TMZ) or doxorubicin.^9,10^ In recent years, liposomal drug delivery systems have emerged as a significant therapeutic nanoplatform tested in clinical trials.^11^ As further examples, the therapeutic efficacy of AGuIX nanoparticles combined with radiotherapy and concomitant TMZ is being evaluated in the NANO-GBM phase I/II clinical trial (NCT04881032), while aminosilane-coated superparamagnetic iron oxide nanoparticles (NanoTherm®) are under investigation in the ANCHIALE study (NCT06271421).^12^

The above-mentioned studies demonstrate the multifaceted nature of GBM research, consequently leading to a growing number of new therapies in the preclinical phase. Additionally, considering that over 90% of new therapies are abandoned in the drug development cycle due to insufficient efficacy or safety profiles, a strong need for refining screening methodologies is emerging: this would enable faster identification and selection of therapies for advancement into clinical trials.^13^

The use of experimental animal models constitutes a crucial stage in the preclinical phase. Animal models play a significant role in understanding the biology of GBM and in developing and testing new therapies. In GBM research, rodent-based models are primarily used;^14^ however, alternative methods are being investigated due to the numerous drawbacks of rodent models, including lack of tumor interaction with the immune system, the absence of or weak tumor heterogeneity, different physiology, high cost, limited reproducibility, and ethical issues. The main alternative is represented by the use of zebrafish and its embryos,^15^ but despite achieving greater throughput, the main disadvantage is represented by high variability and susceptibility to environmental conditions.

There is a pressing need for *in vitro* platforms that not only replicate the 3D complexity of tumor microenvironments but also enable high-resolution, real-time monitoring of drug responses. This remains a major limitation in current preclinical testing pipelines, where the lack of dynamic and spatially resolved readouts impairs the ability to screen and prioritize candidate therapies. Current efforts are focused on finding the right bridge between cell-based research and laboratory animal testing, so as to pursue preclinical research adhering to the “3Rs” principle—reduce, refine, replace—, in order to minimize animal use while upholding scientific integrity and result reliability.^16,17^*In vitro* platforms, notably lab-on-chip systems, offer promising alternatives to traditional animal models, with the FDA directive of December 2022 underscoring the pivotal role of *in vitro* platforms in drug development, potentially eliminating the necessity for animal models.^18^ Incorporating glioma cells into lab-on-chip systems has led to the development of GBM-on-a-chip devices. This approach allows for the formation of an *in vitro* tumor model with histological, biochemical, and physiological complexity characteristic of *in vivo* models. With GBM-on-a-chip devices, we can mimic the pathophysiology and the tumor microenvironment along both time and space.^19^ These models mimic the *in vivo* tumor microenvironment more effectively than 2D cultures, encompassing characteristics like cell–cell interactions, the presence of extracellular matrix (ECM), and formation of gradients of nutrients and oxygen, thereby offering a more realistic depiction of the intricate conditions within a tumor.^20,21^ Moreover, 3D tumoroids show drug responses that better reflect *in vivo* conditions. This is critical for drug testing, as this enables a more precise prediction about how a substance could work within a complex tumor microenvironment: enhanced predictive accuracy can translate into a more successful identification of potential therapeutic candidates.^22,23^

GBM-on-chip systems offer another significant advantage: the ability to assess cytotoxicity in real time through appropriately fabricated measurement electrodes. Impedance spectroscopy analysis stands out as a commonly utilized method for this purpose. Through these measurements, we not only gather data on the potentially toxic effects of the tested substance, but also track the dynamics of cell growth, which is of pivotal importance for the understanding of tumoroid formation. Electrical impedance spectroscopy is a real-time, label-free method for cell behavior analysis, extensively employed in 2D cell cultures. The application of this technique in 3D cellular models presents challenges due to the complexity of the electrical systems; in adherent models, cells attach directly to the electrodes, while in 3D models, they are embedded within specialized 3D matrices: as a result, integrating electrodes into 3D models becomes a significant technical challenge.^24^

In this work, we propose a platform offering the opportunity for multiple measurements of the electrical impedance of a 3D GBM culture, enabling real-time monitoring of drug cytotoxic activity. Moreover, the developed device is sensitive enough to detect small differences in a few hours of treatment. The desired 3D GBM microscopic foci model is achieved by self-organizing GBM cells seeded into a culture chamber, where 3D scaffolds are fabricated by two-photon polymerization (2 pp) and directly placed on the measuring electrodes. The 2 pp approach offers unique benefits over conventional anti-cell adhesion or ultra-low attachment plate methods, which often require complex microfluidic integration for impedance monitoring and typically fail to probe the inner structure of the spheroid. Indeed, the 2 pp technique enables real 3D, high-resolution fabrication of porous and cell-adherent scaffolds that promote stable cell colonization and long-term growth. These microarchitectures can be precisely tailored to ensure optimal positioning with respect to the electrodes, facilitating consistent and spatially resolved impedance signals. Recent work of our group has further demonstrated the capacity of 2 pp scaffolds to guide the 3D cellular architecture with high fidelity and reproducibility, particularly for drug screening.^25,26^ Additionally, this device allows for optical analysis of tumor growth dynamics through a transparent interface, allowing for the analysis of biomarkers using traditional immunocytochemical methods. The innovative design of this platform is further supported by a recently filed patent application (IT102024000016873), where its technological uniqueness and potential for translational impact are highlighted.

## 2. Results & discussion

### 2.1 Design and fabrication of the device

**Fig. 1:**
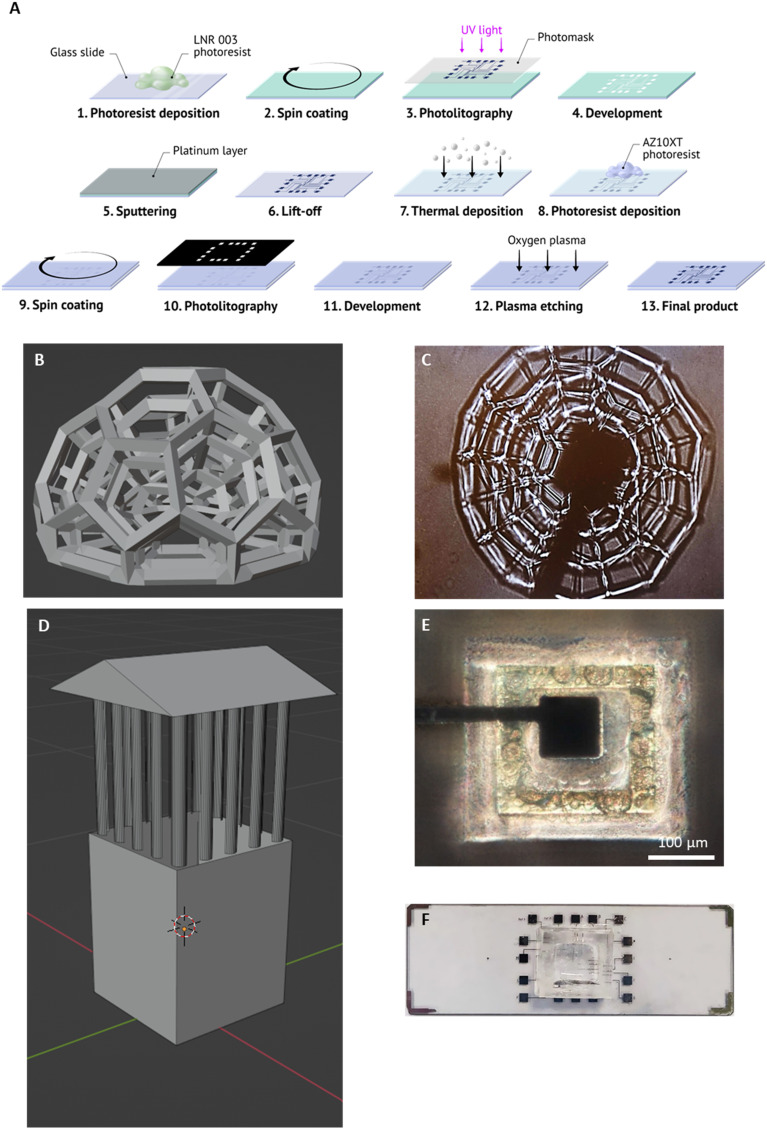
Device for real-time, multiplex monitoring of the growth and drug response of 3D tumor cultures. (A) Procedure for the multielectrode array fabrication on a glass substrate. (B) Design of the multi-layered cupola-like scaffold to promote cell colonization. (C) Microphotograph of a representative cupola-like scaffold fabricated on the measurement electrode using 2 pp. (D) Design of the tower-like scaffold to avoid cell adhesion and colonization. (E) Microphotograph of a representative tower-like scaffold fabricated by 2 pp on the reference electrode. (F) Final device with a PDMS chamber suitable for cell culture, which isolates the electrodes from the conductive pads.

The device has been designed to enable real-time, multiplex monitoring of the growth and cytotoxic response of 3D cell models. A key aspect of this approach involves the fabrication of a multielectrode array (MEA) with planar electrodes, integrated with 3D microscaffolds designed to either promote or inhibit cell colonization, thereby enabling precise spatial control over tumor cell growth for studying biological processes in colonized regions while maintaining cell-free areas for accurate electrical impedance measurements. Fig. 1 shows the design and fabrication of the device. The MEA comprises 16 electrodes obtained by metal deposition of platinum (Fig. 1A), selected due to its high biocompatibility and chemical stability;^27^ parylene C was used as a biocompatible electrical insulator of the conductive paths.^28,29^ Positive and negative photomasks used for metal deposition and plasma etching are reported in Fig. S1.† To enable cell colonization, two distinct 3D microscaffold designs were developed by 2 pp and precisely integrated with the MEA through an automated process (Video S1†). The first design is a concentric multi-layered cupola-like scaffold, shown in Fig. 1B, which is engineered with a porous dome-like architecture to promote the growth of 3D cell clusters (details of the size of the structures and pores are reported in Fig. S2†). Specifically, the pore dimensions were optimized to allow U-87 MG GBM cells (∼50 μm in diameter) to enter and stably adhere within the 3D scaffold during the seeding phase. The larger outer cupola openings ensure accessibility for initial cell entry, while the presence of smaller nested cupolae and internal microcavities supports progressive cell infiltration, interconnection, and tissue compaction over time. This architectural strategy facilitates the formation of highly organized, dense spheroids that remain stably anchored to the sensing area, enabling consistent and reproducible impedance readouts. This specific multi-scale design, which combines open architecture for seeding and internal confinement for tissue self-organization, is a core feature of the invention disclosed in our recent patent application (IT102024000016873). A microphotograph of the fabricated cupola structure on the measurement electrode is presented in Fig. 1C. The second structure is a tower-like scaffold (Fig. 1D) specifically engineered to maintain the functionality of the reference electrodes. The “sloped roof” design prevents cell adhesion during the seeding process, ensuring that no cells settle on the reference electrode as well as on the top of the scaffold. The 392 μm high walls at the base of the scaffold act as an effective barrier, preventing subsequent colonization by cell migration (details on the tower-like scaffold size are provided in Fig. S3†). The microscopy image of the tower-like scaffold surrounding the reference electrode is shown in Fig. 1E. The columns connecting the roof and walls create an open system: this allows electrical impedance to be accurately measured with respect to the measurement electrodes while maintaining the structural integrity of the scaffold. The final fabricated device is shown in Fig. 1F, incorporating a PDMS chamber specifically designed for cell culture, which effectively isolates the electrodes from the conductive pads; the latter ones serve as the interface for operators to perform impedance measurements. To validate the durability for long-term experiments, we conducted impedance stability tests by immersing the device (MEA with 2 pp structures) in cell culture medium for 10 days. This period corresponds to a typical timeframe used for 3D culture formation and subsequent drug treatment analysis. As reported in Fig. S4,† the impedance spectra remained remarkably stable throughout the test. Fig. S4A† displays the full-range impedance profiles over time, while Fig. S4B† shows data at selected frequencies, confirming the absence of significant drift.

Although the 3D design of the microscaffolds was optimized for biological functionality rather than high-throughput manufacturing, the process already demonstrates promising scalability. Functionalization of each MEA with 3D microscaffolds requires approximately 1.5 h per device. Importantly, the 2 pp systems used for scaffold fabrication are capable of overnight operation and can reliably produce up to 16 devices per day per system. Given that the MEA fabrication remains the most time-consuming step in the overall workflow, 2 pp does not represent a bottleneck. Moreover, the cost contribution of the 2 pp step is relatively minor compared to the MEA itself, which includes high-precision electrode patterning and encapsulation. These considerations underscore the feasibility of scaling up production and support the translational potential of the device for broader use in preclinical research. The device is user-friendly and requires minimal training: impedance measurements are obtained by simply connecting the electrode pads to an external analyzer. This straightforward operation eliminates the need for complex biological processing steps. Furthermore, future versions of the device could incorporate a multi-pin connector interface to enable fully automated, plug-and-play integration.

### 2.2 Impedance response in 2D and 3D cell cultures

**Fig. 2:**
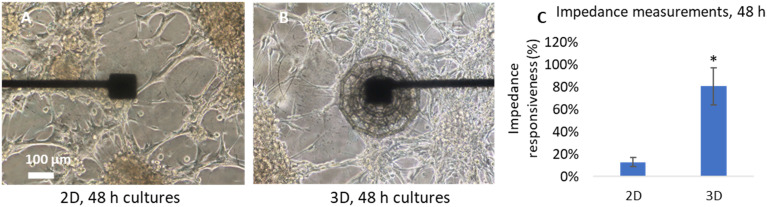
Comparison between 2D and 3D U-87 MG cultures on electrodes. (A) Representative brightfield image of a 2D culture. (B) Representative brightfield image of a 3D cell cluster developed on the scaffold at 48 h of culture. (C) Percentage of responsive electrodes in 2D and 3D configurations, showing a significant change in impedance at 48 h of culture; * *p* < 0.05.

Impedance measurements were performed using a platinum reference electrode positioned in the center of the well. The remarkable differences in impedance measurements between 2D and 3D cell cultures highlighted the critical role of the multi-layered cupola-like architecture in achieving cell colonization (Fig. 2). Brightfield images of U-87 MG cells at 48 h of culture reveal distinct behaviors under 2D and 3D conditions; under 2D conditions (Fig. 2A), cells spontaneously formed random clusters but rarely remained stable on the planar electrode surfaces. As a result, only a small percentage of measurement electrodes (13 ± 4%; *n* = 16) showed significant impedance increases compared to the controls without cells. Furthermore, the inherent migration of clusters during the culture period introduces complexity to the analysis; thus, the observed impedance changes may stem from cluster movement rather than genuine cellular responses to treatments. Conversely, the introduction of multi-layered cupola-like scaffolds significantly enhances measurement reliability. U-87 MG cells cultured under 3D conditions efficiently colonized the scaffold surfaces and formed spheroids localized around the measurement electrodes at 48 h of culture (Fig. 2B). This arrangement led to a markedly higher percentage of responsive electrodes (81 ± 10%; *n* = 26) across the array, as shown in Fig. 2C.

These results highlight the advantages of 3D culture systems over 2D configurations, demonstrating their ability to better replicate *in vivo*-like environments while enabling accurate and reproducible impedance-based measurements. In traditional impedance-based cytometry, microfluidic platforms have been designed to analyze single cells. For example, Zhong *et al.* introduced a microfluidic-based method for label-free, high-throughput impedance measurements of single-cell viability.^30^ Their system also exploited complex “opacity” spectrum analysis, a novel approach developed by Zhong *et al.* to distinguish live from dead cells with high accuracy in a two-dimensional setup. This method involved integrating the phase of single cells with opacity, a parameter defined as the ratio of high-frequency to low-frequency impedance magnitude, which is used to infer the dielectric properties of the cells. As another example, Yang *et al.* recently presented an impedance cytometry device for analyzing cell viability in a 2D microfluidic environment.^31^ Their approach also focused on distinguishing between live and dead cells using impedance changes, with a focus on indium tin oxide (ITO) electrodes. While effective for single-cell viability in a controlled 2D environment, such systems lacked the ability to mimic *in vivo*-like 3D growth and drug responses. Compared to other impedance-based or microfluidic platforms, the proposed system offers key advantages for 3D tumor modeling and drug screening. Conventional microfluidic impedance systems, such as those developed by Zhong *et al.*^30^ and Yang *et al.*,^31^ are optimized for high-throughput analysis of single cells in 2D environments. These setups often rely on suspended cells and channel-based flow systems, limiting their ability to mimic complex 3D interactions. In contrast, our platform combines real-time impedance monitoring with spatially organized 3D scaffolds, enabling long-term culture and tracking of tumor spheroids *in situ*. Most importantly, our scaffold design allows cells to grow directly on the sensing electrodes, enabling impedance measurements that reflect changes occurring from within the spheroid core. This internal sensing capability offers a more accurate representation of cytotoxic responses in 3D models, which are typically inaccessible to conventional approaches. Furthermore, while systems based on indium tin oxide (ITO) or gold electrodes may lack stability in long-term cultures, the use of platinum electrodes integrated with biocompatible parylene C insulation ensures durability and signal fidelity, as validated by the impedance stability tests (Fig. S4†). The scaffold-integrated reference electrode further enhances measurement consistency, avoiding manual repositioning and user variability typical of systems with external references. Collectively, these features position our platform as a robust and scalable solution for multiplexed, label-free assessment of drug responses in 3D cell models, offering distinct benefits over conventional impedance cytometry approaches.

### 2.3 Impedance changes over time and in response to drug treatments

**Fig. 3:**
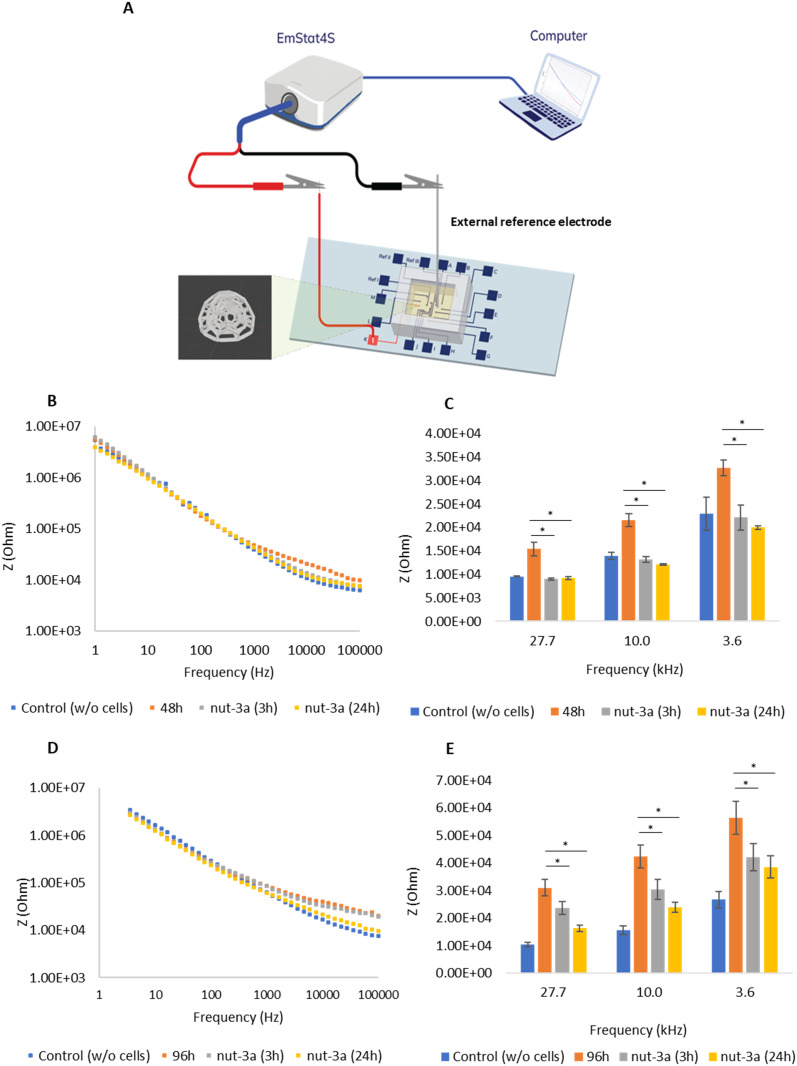
(A) Schematic of electrical impedance measurement using an external electrode. (B) Changes in electrical impedance across the full frequency range at 48 h of culture under 3D conditions and (C) for selected frequency values in response to 30 μM nut-3a treatments (3 and 24 h). (D) Changes in electrical impedance in the 96 h-3D cultures across the full frequency range and (E) for selected frequency values. * *p* < 0.05.

Following the analysis of colonization dynamics in 2D and 3D systems, we investigated how electrical impedance responded to the nutlin-3a (nut-3a) anticancer drug in both 48 h- and 96 h-grown spheroids. These results, presented in Fig. 3, illustrate device capacity to detect real-time changes in cell growth and treatment responses. Impedance measurements were obtained using a platinum external reference electrode immersed in the culture medium, in combination with the scaffold-integrated measurement electrode (Fig. 3A). Regarding experiments on 48 h cultures, impedance values recorded across the entire frequency spectrum (Fig. 3B) and average impedances calculated at high frequencies (27.7 kHz, 10.0 kHz, and 3.6 kHz; Fig. 3C) showed significant reductions following 30 μM nut-3a treatment. Both 3 h and 24 h nut-3a treatments led to a pronounced decrease in impedance, with values approaching those of plain electrodes without cells. This suggests substantial cell death or detachment, following cytotoxic stress.^30^ In contrast, the 96 h cultures (Fig. 3D and E) demonstrated a progressive decrease in impedance post-treatment (30 μM nut-3a), though these values did not return to pre-seeding levels. This observation suggests that higher drug concentrations or extended treatment times may be required to completely eradicate 96 h tumor spheroids, therefore highlighting the utility of the platform for screening how drug responses vary along the maturation of 3D models. SEM images of 96 h 3D cultures further confirm spheroid formation and degradation upon post-nut-3a treatment (Fig. S5†).

To study the dose-dependent cytotoxic effects of nut-3a in 3D GBM spheroids, we performed impedance-based analysis using three additional drug concentrations below 30 μM (Fig. S6†): 10 μM, 15 μM, and 20 μM. These tests were carried out on 96 h matured 3D cultures, a timepoint selected for the more advanced tumor model architecture and lower drug sensitivity. Fig. S6A† presents the impedance spectra across the full frequency range following nut-3a treatment for each concentration, while Fig. S6B† highlights impedance changes at selected frequencies. The results demonstrated that 10 μM and 15 μM nut-3a concentrations were insufficient to induce a significant reduction in impedance at 24 h post-treatment (*p* > 0.05). These concentrations prevented the impedance increase over time, suggesting a cytostatic rather than a cytotoxic effect. In contrast, 20 μM nut-3a produced a significant drop in impedance, indicating marked cytotoxicity (*p* < 0.05), though impedance values did not return to pre-seeding levels as for 30 μM nut-3a treatment in 96 h spheroids. This trend was observed both in response to short-term (3 h; −30% at 3.6 kHz, −25% at 10.0 kHz, and −16% at 27.7 kHz) and extended (24 h; −47% at 3.6 kHz, −44% at 10.0 kHz, and −36% at 27.7 kHz) treatments, confirming the potency of the 20 μM dose in overcoming the drug sensitivity threshold. Fig. S6C† reports the cell index (CI), which is the normalized impedance of treated spheroids relative to the pre-treatment control spheroids, for each concentration and treatment duration. Just the 20 μM conditions produced a significant and persistent CI reduction, as observed with the 30 μM treatment.

These findings highlight the importance of dosage when evaluating drug effects in 3D models and confirm that GBM spheroids exhibit decreased nut-3a sensitivity compared to 2D cultures, consistent with previous studies highlighting the barrier-like properties of 3D tumor architectures.^31,32^ The sensitivity of our system is clearly demonstrated by this test, where a modest concentration change from 15 μM to 20 μM resulted in a marked and statistically significant reduction in impedance and the CI. This ability to detect biologically meaningful differences with relatively small variations in drug concentration demonstrates the sensitivity of our approach.

**Fig. 4:**
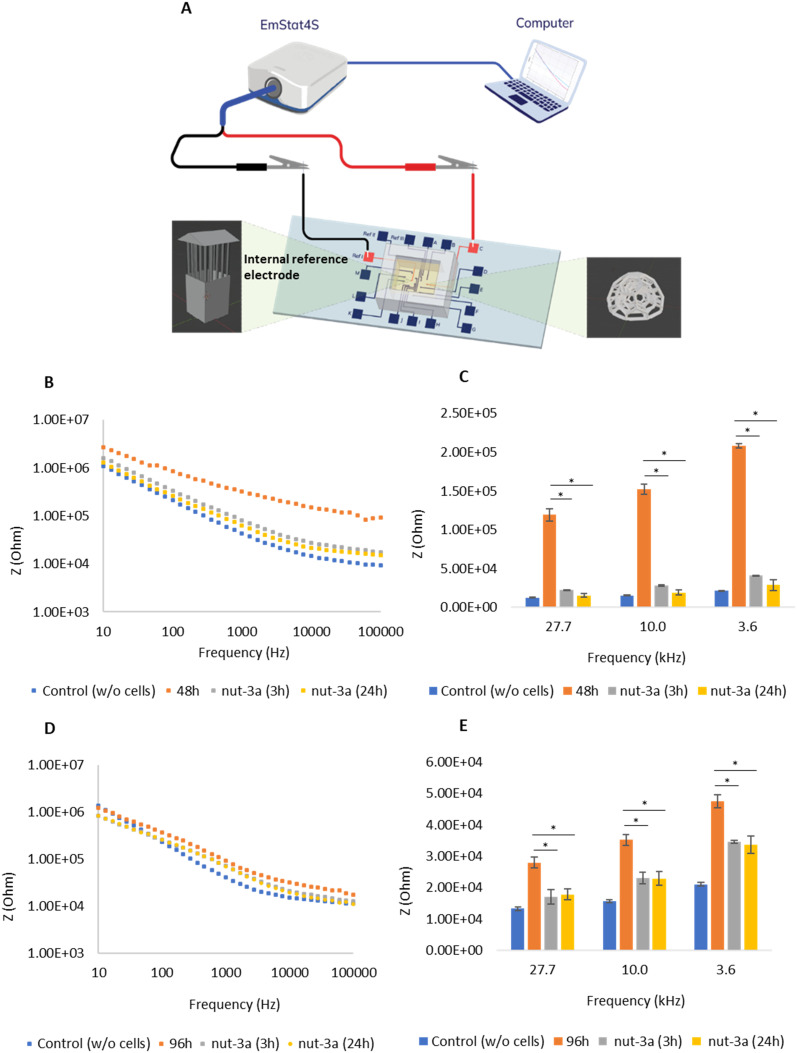
(A) Schematic of electrical impedance measurement using the internal reference electrode equipped with a tower-like scaffold. (B) Changes in electrical impedance across the full frequency range at 48 h of culture under 3D conditions and (C) for selected frequency values in response to 30 μM nut-3a treatments (3 and 24 h). (D) Changes in electrical impedance in the 96 h-3D cultures across the full frequency range and (E) for selected frequency values. * *p* < 0.05.

To validate the functionality of the platform, we conducted additional impedance measurements (Fig. 4) using the integrated reference electrode system equipped with the protective tower-like scaffold (Fig. 4A). This setup ensures isolation from undesired cell adhesion on reference electrodes, improving reliability and avoiding manual measurement by positioning an external electrode in the medium. Impedance data were collected from 3D tumor spheroids cultured for 48 h (Fig. 4B and C) or 96 h (Fig. 4D and E), and subsequently treated with nut-3a 30 μM for 3 and 24 h. Similar to results obtained with the external reference electrode (Fig. 3), we observed a significant decrease in impedance values following drug treatment for both culture “age”, consistent with a cytotoxic response. Also in this case, a progressive increase in anticancer effects was observed in 96 h spheroids from 3 to 24 h nut-3a treatment. The integration of the internal reference electrode system allows for automatic measurements to be performed, with a consequent reduced user variability, a particular critical issue in high-throughput drug screening applications. Overall, the data confirm the robustness of the platform in monitoring spheroid development and drug-induced impedance changes under standardized conditions, supporting its applicability in high-throughput drug screening assays.

**Fig. 5:**
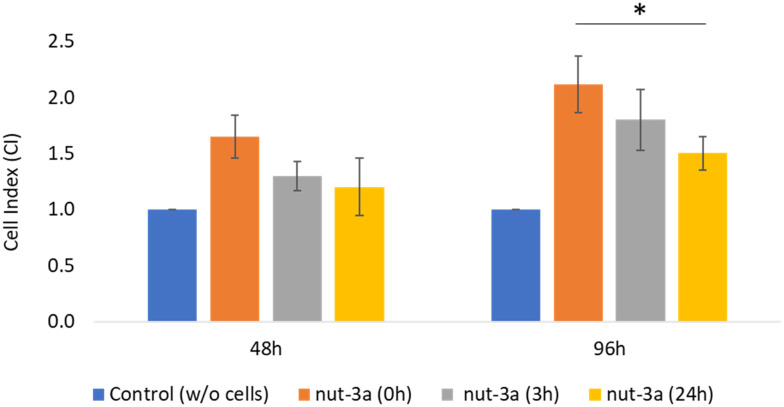
CI in 48 h and 96 h treated spheroids incubated with nut-3a for 3 and 24 h. * *p* < 0.05.

To quantify the tumor growth and response to drug treatment, we calculated the CI. As shown in Fig. 5, CI values increased over culture time, with 96 h cultures displaying higher CI values compared to 48 h cultures. Nut-3a treatment led to a decrease in the CI for both culture durations. For 48 h cultures, CI values approached control levels at 3 and 24 h of treatment, indicating extensive cell death. However, the CI decrease in 96 h cultures was lower, especially for the 3 h treatment, suggesting reduced drug sensitivity.

This trend aligns with studies in other 3D tumor models, such as SKOV-3 spheroids, where reduced drug responsiveness was observed with prolonged culture durations.^33^ These findings underline the potential of the platform in assessing dynamic changes in drug sensitivity in 3D tumor models without the need for biological assays, providing a robust platform for real-time, multiplexing, and quantitative pharmacological analysis. To date, there is no evidence of systems allowing the monitoring of impedance values in 3D glioma models; however, analogous studies have been carried out on other systems, such as A549 spheroids, and confirm that impedance values increase along with the culture time and are sensitive to cytotoxic treatments^34^ in a dose- and time-dependent manner. This work also demonstrates the validity of introducing the CI value; according to its definition, this is the ratio of the impedance change caused by the growth of 3D cells to the background impedance, and it is useful in describing phenomena such as cancer cell proliferation and apoptotic response.^35^

### 2.4 Biological testing for system validation

**Fig. 6:**
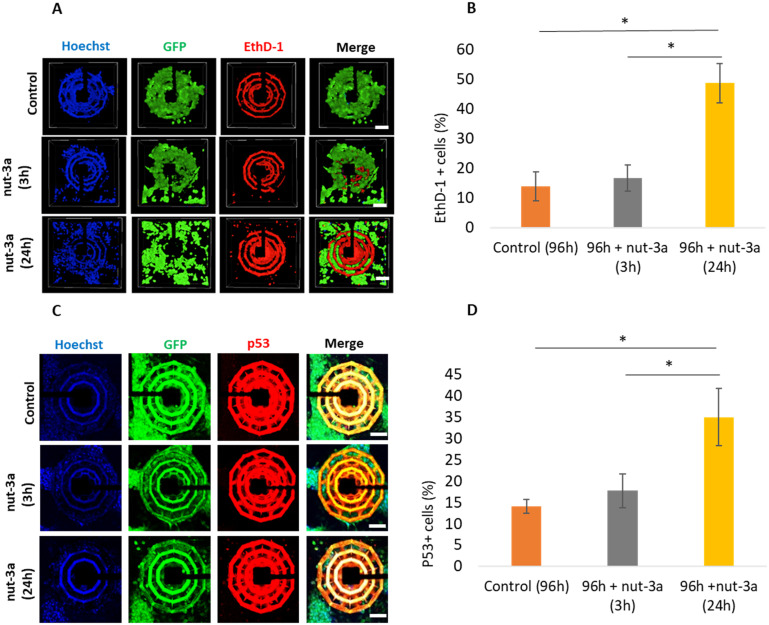
Biological validation on 96 h spheroids undergoing nut-3a treatment. (A) Representative fluorescence images of dead cells (EthD-1 in red; cytoplasmic GFP in green; nuclei in blue). (B) Graph reporting EthD-1^+^ cells (%). (C) Representative fluorescence images of p53^+^ apoptotic cells (p53 in red; cytoplasmic GFP in green; nuclei in blue). (D) Graph reporting p53^+^ cells (%). * *p* < 0.05.

To confirm whether the observed impedance reduction corresponds to cell death, we conducted biological validation experiments (Fig. 6), specifically conducted on 96 h spheroids, where a progressive increase in the drug-dependent cytotoxic effects was observed with longer exposure to nut-3a 30 μM. EthD-1 staining (confocal images reported in Fig. 6A), which marks membrane integrity loss, revealed no significant changes at 3 h of treatment (16.6 ± 4.4% EthD-1^+^ cells in treated cultures *vs.* 13.8 ± 4.9% in the control). However, after 24 h of treatment, EthD-1^+^ cells significantly increased to 48.6 ± 6.7%, indicating extensive cell death (Fig. 6B). Apoptosis was moreover assessed by evaluating p53 marker expression. Confocal imaging (Fig. 6C) and quantitative analysis (Fig. 6D) showed no substantial rise in p53^+^ cells at 3 h of treatment (17.7 ± 4.0% in treated *vs.* 14.6 ± 2.0% in the control). At 24 h, however, p53^+^ cells significantly increased to 35.6 ± 6.8%.

The biological validation performed on 96 h spheroids strongly supports the reliability of impedance measurements as an effective indicator of cell death. The significant increase in EthD-1^+^ and p53^+^ cells at 24 h of 30 μM nut-3a treatment correlates well with the observed reduction in impedance, confirming the sensitivity of the system in detecting cytotoxic effects. Interestingly, our platform was able to detect a decrease in impedance at 3 h of treatment, even when cellular death was not immediately visible through conventional staining methods. This discrepancy may be attributed to cell detachment from the spheroids, which may hinder cell death detection through standard immunostaining techniques, due to the loss of cells from the outer layers of the spheroids. Traditional assays like EthD-1 staining and p53 marker analysis can detect cell death within the spheroids but may underestimate its extent, particularly when cells detach; conversely, the impedance measurement detects changes across the entire spheroid structure. Its ability to continuously monitor impedance during cell detachment offers a measure of the overall cell viability; additionally, it is worth mentioning that the optical transparency of the platform allows performing traditional assays, including immunofluorescence. This feature enables integration of optical and electrical data, providing a better understanding of cell behavior and improved pharmacological evaluations.

### 2.5 Necrotic core detection

One of the hallmarks of spheroids, especially those cultured for extended periods, is the development of a necrotic core. This phenomenon occurs due to the limited diffusion of nutrients and oxygen within the dense 3D structure, resulting in cell death at the core level.^36^ Detecting and quantifying this necrotic region is crucial in cancer research and drug testing. Indeed, necrotic cores are associated with tumor growth and resistance to treatments, as cells surviving in these regions are often more resistant to chemotherapeutic agents due to poor drug penetration and a lack of oxygen.^37^

**Fig. 7:**
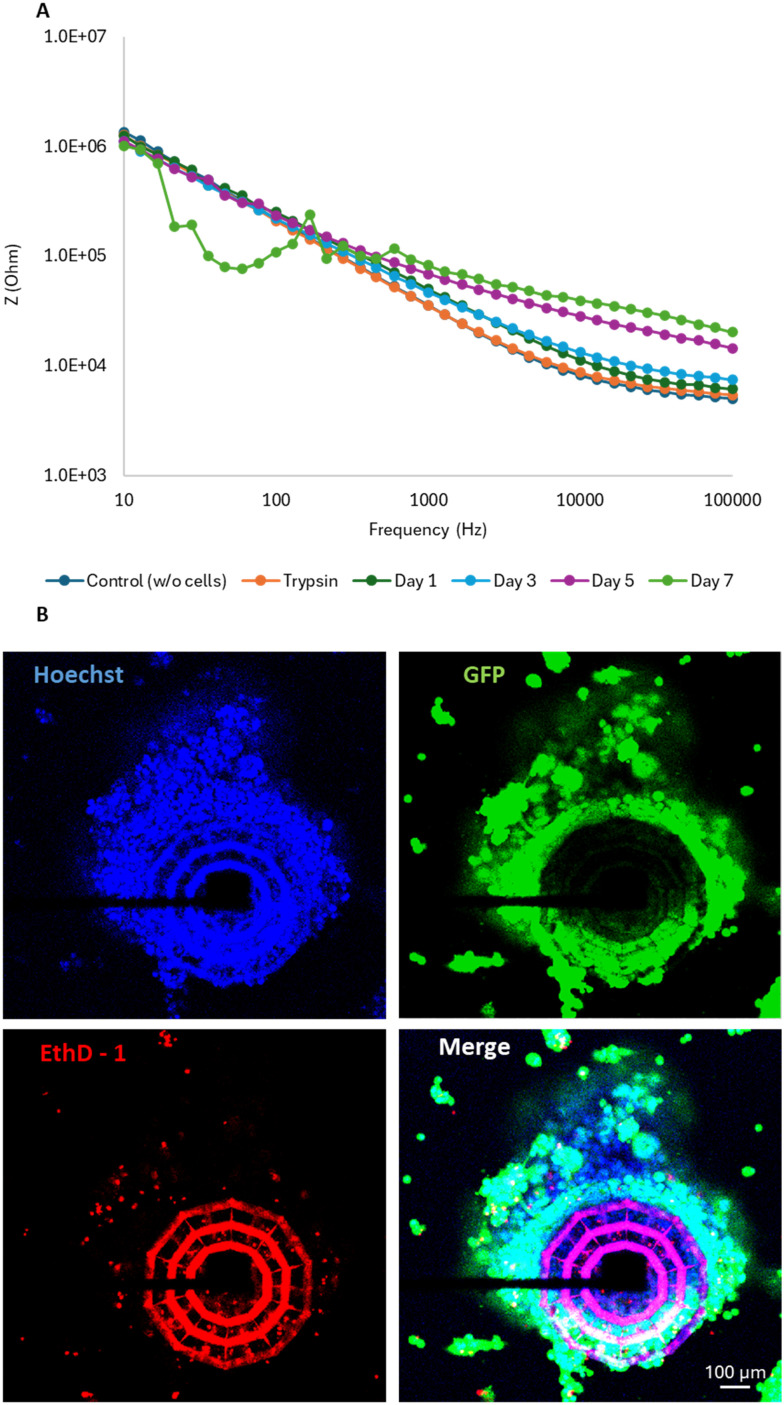
Study of extended cultures on 3D scaffolds. (A) Representative impedance measurements at various frequencies for spheroids over 0–7 days. (B) Representative confocal fluorescence images of necrotic cells. A white outline has been added to the “Merge” image to delineate the boundaries of the necrotic core.

In this work, we aimed to investigate the formation of the necrotic core in spheroids over extended culture periods and explore the potential of electrical impedance measurements as a non-invasive tool for detecting this phenomenon (Fig. 7). Impedance measurements were conducted at multiple time points on 20 spheroids. As illustrated in Fig. 7A, the electrical impedance exhibited a progressive increase over the course of culture at frequencies ranging from approximately 100 Hz to 100 kHz. Notably, values tended to converge at lower frequencies, which was consistent with the expected impedance behavior for spheroids cultured for 48 and 96 h. However, in the extended culture model (on day 7), impedance values significantly decreased at low frequencies (∼10 to ∼100 Hz), accompanied by disruptions in signal conduction (Fig. 7A, green trace). To corroborate these findings, EthD-1 staining was performed to assess cell viability within the spheroids. The staining revealed pronounced EthD-1 internalization, predominantly in the core region (Fig. 7B), indicating cell death. These results suggest that the observed impedance changes were closely linked to the development of an internal cavity and to the accumulation of non-viable cells within the spheroid core. This pattern reflects significant cell death concentrated within the spheroid cavity. The observed decrease in impedance may arise from structural changes in the spheroid, such as modified conductivity due to reduced viability. Disruptions in signal conduction can be attributed to the strong heterogeneity in cell density within the spheroids, with regions where cells are densely packed, especially in the outer layers, and the core that undergoes necrosis. Specifically, this heterogeneity can affect impedance measurement: the dense outer layers can act as an insulating barrier, causing current to flow around the cells *via* paracellular pathways^38^ and increasing impedance at high frequency, while necrotic regions with fewer viable cells lead to reduced impedance, especially at lower frequencies. Importantly, this effect was reversible and was not associated with electrode failure: after trypsinization, which removed the cells, impedance values returned to levels comparable to the control (Fig. 7A, orange trace), confirming that the observed changes originated from spheroid behavior and not from technical artifacts.

To perform a better interpretation of the frequency-dependent features of the impedance spectra, we considered the biophysical mechanisms underlying the signal across different frequency ranges. In our study, the observed frequency-dependent impedance profiles reflect the combined contributions of both the extracellular microenvironment and the intracellular properties of the 3D spheroids. At low frequencies, the electric field is largely restricted to the extracellular space due to the insulating nature of intact cell membranes.^39^ Consequently, impedance in this range is mainly influenced by cell-to-cell contacts, extracellular matrix organization, and overall spheroid cohesion. A reduction in impedance at these frequencies typically indicates loss of structural integrity, such as cell detachment or weakening of intercellular junctions.^6^ In contrast, at higher frequencies, the electric field can partially penetrate cell membranes, making impedance sensitive to intracellular conductivity and membrane integrity.^40^ Therefore, changes at these frequencies can reflect apoptotic or necrotic processes, such as membrane permeabilization and cytoplasmic reorganization. These effects are consistent with our findings following 30 μM nut-3a treatment, which induced impedance reductions across the entire spectrum, along with both cell detachment and increased EthD-1^+^/p53^+^ cell populations.

### 2.6 Evaluation of temozolomide responses

Temozolomide (TMZ) is a DNA-alkylating agent and the current standard-of-care for GBM therapy.^41^ Using our impedance-based system, we assessed cytotoxicity in U-87 MG spheroids following a single administration of 30 mM TMZ (Fig. S7†). At 24 h post-treatment, the CI showed a slight but statistically significant reduction of approximately 5.7% compared to the pre-treatment baseline. By 48 h, the CI has further decreased by about 11.5%. These progressive declines indicate the onset and gradual intensification of drug-induced cytotoxic effects. These findings are consistent with flow cytometry analysis performed on spheroids cultured under non-adherent conditions, which revealed a 13.3% increase in annexin-V^+^ apoptotic cells when treated for 48 h with 30 μM TMZ, compared to untreated controls (Fig. S7D†).

The agreement between the two independent readouts highlights the reliability of our impedance-based platform. U-87 MG cells are generally considered TMZ-sensitive,^42^ consistent with our findings showing a significant cytotoxic effect at elevated TMZ concentrations. However, our platform revealed that higher doses were required to elicit detectable responses in 3D cultures compared to traditional 2D models.^42^ This likely reflects the impact of the 3D microenvironment, such as limited drug diffusion, enhanced cell–cell and cell–matrix interactions, and altered metabolic gradients, which can modulate drug accessibility and efficacy.^43^ These observations reinforce the critical role of 3D culture systems in recapitulating physiologically relevant tumor responses, and underscore the sensitivity of our impedance-based platform in monitoring such dynamics.

### 2.7 Evaluation of sorafenib responses

To evaluate the ability of the device to assess drug responses in non-neural tumor types, we investigated the cytotoxic effects of sorafenib (SORA) on CaCo-2 colorectal adenocarcinoma spheroids. At 96 h of culture, the 3D spheroids were treated with 15 μM SORA, and impedance values were recorded after 3 and 24 h of exposure (Fig. S8†). Fig. S8A† presents the impedance spectra across the full frequency range, Fig. S8B† highlights impedance changes at selected frequencies (3.6 kHz, 10 kHz, and 27.7 kHz), and Fig. S8C† reports the calculated CI for each treatment condition. A progressive decline in CI values was observed, with a statistically significant reduction (∼37%; *p* < 0.05) at 24 h post-treatment, demonstrating the platform ability to detect pharmacological effects in 3D colorectal cancer models as well.

These results extend the applicability of our platform beyond GBM, and demonstrate its suitability for analyzing pharmacological responses in epithelial tumors. The sensitivity of the device to detect even early impedance shifts confirms its potential as a real-time, label-free monitoring system applicable to a wide range of drug mechanisms, including kinase inhibitors like SORA.

Importantly, these experiments support the versatility of the device in several aspects: i) the system successfully hosted CaCo-2 cells, indicating compatibility with different tissue origins and morphologies; ii) the platform detected the effects of a multi-kinase inhibitor, in addition to the p53-MDM2 antagonist (nut-3a) and DNA-alkylating agent (TMZ), showcasing its broad detection spectrum; iii) the CI measurement offers a standardized, easy-to-compare metric of cell behavior under treatment, supporting high-throughput and multiplexing applications. Altogether, these findings emphasize the robustness and versatility of our impedance-based platform as a powerful tool for preclinical drug screening; its ability to function across multiple tumor types and drugs, including agents with different mechanisms of action, positions it as a strong candidate for use in personalized medicine and precision oncology pipelines.

## 3. Conclusion

In this study, we developed a GBM-on-chip model particularly suitable for the evaluation of tumor spheroid development, sensorized for impedance measurements. This platform enabled real-time, non-invasive, and label-free analysis of GBM microscopic foci-mimicking tumor spheroids and provided critical insights into tumor growth dynamics and therapeutic responses. Our results reveal that the platform is able to track impedance changes associated with spheroid maturation and drug treatments, including subtle variations that can be missed by conventional biological assays (*e.g.*, when cell death is accompanied by cell detachment). Furthermore, the optical transparency of the platform facilitated integration with immunofluorescence and confocal microscopy, allowing for multimodal data acquisition and more detailed characterization of cellular behaviors. Compared to existing systems,^30–32,44^ the proposed platform offers significant advantages, including the elimination of microfluidic requirements, simplified setup, and compatibility with multiplexing high-throughput screening formats. The developed microphysiological system thus represents a multi-tasking and innovative tool, contributing to bridging the gap between *in vitro* and *in vivo* research.

Future studies could include a broader range of tumor spheroid models, such as patient-derived organoids and co-culture systems that incorporate stromal and immune cells. This would enhance the physiological relevance of the platform for studying tumor microenvironments and therapeutic responses. Furthermore, the combination of impedance data with proteomics, transcriptomics, and metabolomics could provide a deeper understanding of the molecular mechanisms underlying spheroid growth and drug responses. Moreover, while this study focused on tumor models, research may also be directed towards other applications, such as performing (nano)cytotoxicity investigations on highly biomimetic 3D models.

The integration of softer and more compliant materials will also be considered to more closely replicate the biomechanical properties of the brain (or, in general, of soft tissues) microenvironment. The incorporation of hydrogels and/or alternative biocompatible polymers with lower stiffness could enable more physiologically relevant cell behavior, thus improving the predictive power of drug response assays. Recent efforts in this direction have demonstrated the benefit of soft matrices in recapitulating the mechanobiology of the brain, with direct implications for cell invasion, proliferation, and therapeutic resistance.^45^ Such approaches would enhance the platform biomimicry and further broaden its translational impact in neuro-oncology.

Eventually, while the presented platform offers high sensitivity, non-invasiveness, and compatibility with 3D spheroid cultures, certain limitations must be acknowledged. Specifically, the impedance-based readout inherently captures an ensemble average of electrical properties across the entire spheroid. As such, it does not resolve single-cell level responses, which may limit its ability to detect subtle heterogeneities or early-stage cell fate changes within a multicellular structure. Future work will aim to integrate complementary high-resolution techniques, such as single-cell imaging and localized biosensors, to dissect single-cell responses. Regarding long-term measurements, we did not observe any noticeable degradation of the scaffold structure over the experimental timeframe: this stability is primarily attributed to the inherent chemical and mechanical robustness of SU-8, which supports the platform potential for repeated use and long-term applications. Finally, although we did not include equivalent circuit modeling in this work, we recognize its potential to further dissect the relative contributions of specific components, and we plan to incorporate such modeling in future studies to strengthen mechanistic interpretation and support broader comparative analysis.

## 4. Experimental section

### 4.1 Fabrication of the device for 3D culture monitoring

Electrodes were fabricated on a glass substrate measuring 76 × 26 × 1 mm^3^ using a multi-step process. The substrate was cleaned using deionized water, acetone, and isopropanol, and dried with an air gun. Subsequently, the glass surface was activated with oxygen plasma for 15 s at 40 W. For the conductive path and electrode fabrication, 2 ml of AZ LNR-003 photoresist was deposited at the center of the glass substrate, which was then placed in a rotating disk. The photoresist spinning was carried out at 2300 rpm for 35 s; subsequently, the glass underwent a controlled heating process on a plate at 120 °C for 2 min.

Two photomasks (Fig. S1a and b†), designed with LibreCAD, were used for metal deposition and plasma etching, respectively. Alignment with photomask A was achieved using a mask aligner, followed by UV exposure at 350 mJ cm^−2^ to pattern the microelectrode design. Post-exposure bake was conducted for 90 s at 100 °C. Development was carried out using an AZ 726 MIF developer for 60 min. A following rinsing step with deionized water was carried out before drying. A 10 nm adhesion layer of Ti and a 150 nm layer of Pt were sputter-deposited on the substrate, using argon as sputtering gas (Ti: argon pressure = 7 × 10^−2^ mbar, 90 W, 0.4 A; Pt: argon pressure = 7 × 10^−2^ mbar, 70 W, 0.25 A). The photoresist was lifted off with acetone, rinsed with isopropanol, and then dried.

The resulting device was further coated with a 500 nm layer of parylene-C by thermal deposition using the PDS 2010 LABCOTER SCS system (furnace: 690 °C, chamber gauge: 135 °C, vaporizer: 175 °C). Two ml of positive photoresist AZ 10 XT was then deposited, spin-coated (35 s, 3000 RPM, 500 RPM s^−1^), and heated for the soft-bake process (2 min at 110 °C). Alignment with photomask B was achieved using a mask aligner, with UV exposure at a dose of 1500 mJ cm^−2^. Development was carried out using an AZ 726 MIF developer for 5 min, followed by rinsing with deionized water and drying with a stream of N_2_. The non-protected parylene-C part was etched with oxygen plasma for 5 min at 100 W. The photoresist was stripped with acetone, and the device was rinsed with isopropanol before final drying.

Both the cupola-like and tower-shaped scaffolds were designed using the Blender software. The 2 pp fabrication process of the 3D scaffolds was performed with non-toxic photoresist IP-S (Nanoscribe GmbH) with a Photonic Professional system (Nanoscribe GmbH). The central part of the device, where the measurement electrodes are located, was coated with a drop of photoresist IP-S, and the objective lens (25×, NA 0.8) of the instrument was immersed in the photoresist. The scaffolds were produced with a writing distance of 0.5 μm, a cutting distance of 1 μm, a writing speed of 10 mm s^−1^, and a laser power of 20 mW. The development was performed using propylene glycol methyl ether acetate (PGMEA) for 10 min, followed by a washing step with isopropyl alcohol for 15 min.

### 4.2 Preparation of the PDMS chamber and stability testing of impedance electrodes

SYLGARD™ 184 Silicone Elastomer Base and SYLGARD™ 184 Silicone Elastomer (Dow) were weighed and combined in a weight ratio of 10 : 1. The mixture underwent heating for 3 h at 75 °C until complete polymerization. Subsequently, a cube with a base area of 1 cm^2^ was cut out with a scalpel. Following this, the same mixture of the elastomer components was then applied as a thin layer around the entire perimeter of the PDMS chamber base and carefully fixed to the glass substrate. Heating for 2 h at 75 °C followed. Then, the chamber interior was filled with fetal bovine serum (FBS) to promote cell adhesion to the glass surface and to the 3D scaffolds, followed by incubation at 37 °C for 3 h.

To evaluate the long-term stability of the impedance electrodes, devices were immersed in complete medium and maintained at 37 °C in a humidified incubator for a period of 10 days. Impedance spectra were recorded using a PalmSens EmStat4S potentiostat impedance analyzer with the PSTrace 5.9 interface and an external platinum electrode centrally immersed in the culture medium at multiple time points (days 1, 4, 7, and 10). The electrodes were not seeded with cells during this test to isolate and assess any medium-induced effects on the device materials.

### 4.3 Cell culture

GFP-expressing U-87 MG cells (Cellomix) were cultured using high-glucose Dulbecco's modified Eagle's medium (DMEM, Gibco) supplemented with 2.5 mM l-glutamine, 25 mM HEPES, 10% heat-inactivated FBS (Gibco), and 1% penicillin–streptomycin (100 IU ml^−1^ penicillin and 100 μg ml^−1^ streptomycin, Gibco). For cell passaging, the cell culture medium was removed, and cells were washed twice with Ca^2+^ and Mg^2+^-free PBS (Sigma-Aldrich) and incubated for 5 min with trypsin (Sigma-Aldrich) before centrifugation and re-seeding. The medium during culture was changed every two days. For the experiments, U-87 MG cells were seeded onto the device into a PDMS chamber with a surface area of 1 cm^2^, at a density of ∼3.5 × 10^4^ cells per cm^2^.

### 4.4 Impedance measurement with cells

The electrical impedance measurements with cells were conducted using a PalmSens EmStat4S potentiostat impedance analyzer with the PSTrace 5.9 interface. Measurements were performed with an external platinum electrode centrally immersed in the culture medium or with an internal reference electrode protected by a tower-shaped scaffold. Control impedance values were obtained before cell seeding. For 2D *vs.* 3D culture experiments, the % of responsive electrodes was calculated as the % of electrodes where a significant change in impedance was found at 48 h of culture.

For nut-3a treatment experiments, impedance measurements were taken on 3D GBM models at 48 and 96 h of culture. Following these measurements, both 48 and 96 h spheroids were treated with 30 μM nut-3a (Sigma Aldrich), and the cytotoxic effect of the drug was monitored at 3 and 24 h of treatment by performing additional impedance measurements.

To evaluate the dose-dependent response of 3D GBM spheroids to nut-3a, we conducted additional experiments using three concentrations: 10 μM, 15 μM, and 20 μM. U-87 MG cells were seeded on the 3D microscaffold-integrated MEA platform and cultured for 96 h to allow spheroid formation. The drug was administered to spheroids for two treatment durations: 3 and 24 h. Electrical impedance was recorded immediately before treatment (baseline) and post-treatment at regular intervals, using a multi-frequency readout (10 Hz–100 kHz). Impedance changes were analyzed across the full spectrum and at selected frequencies (3.6 kHz, 10.0 kHz, 27.7 kHz), and the CI was calculated as previously described.

Concerning TMZ treatments, U-87 MG cells were cultured in the 3D scaffold-integrated device for 96 h, after which TMZ (30 mM) was added to the medium. Impedance measurements were recorded before treatment and at 24 and 48 h of drug exposure. Each condition was tested on a minimum of five devices, and impedance changes were analyzed both across the full frequency range and at selected frequencies of interest (3.6 kHz, 10 kHz, and 27.7 kHz). The CI was then calculated.

To further assess the versatility of our platform across different tumor models, we used CaCo-2 colorectal adenocarcinoma cells (ATCC), a well-established model in gastrointestinal cancer research. After seeding CaCo-2 cells at a density of ∼3.5 × 10^4^ cells per cm^2^ into the 3D scaffold-integrated device and incubating for 96 h to allow spheroid formation, cultures were treated with 15 μM SORA for either 3 or 24 h. Electrical impedance was recorded before and after treatment. Changes in impedance were analyzed across the full frequency range and at selected frequencies (3.6 kHz, 10 kHz, and 27.7 kHz). Finally, the CI was calculated.

### 4.5 Cellular staining

GFP-expressing U-87 MG cells were stained with Hoechst 33342 (1 : 1000, Thermo Fisher Scientific) and ethidium homodimer-1 (2 μM, Thermo Fisher Scientific) for 30 min at 37 °C in cell culture medium, and thereafter rinsed in PBS before incubation with HEPES-supplemented phenol red-free medium for imaging (C2s confocal microscope, Nikon). 3D image reconstruction was performed by using NIS Elements Software (Nikon). For p53 detection, cells were fixed in 4% paraformaldehyde (PFA) for 30 min at 4 °C and permeabilized with 0.1% Triton X-100 in PBS (1 h at 37 °C). After a step in a blocking solution (10% goat serum in PBS, 1 h incubation; Invitrogen), samples were subjected to immunostaining through incubation with a primary mouse anti-p53 antibody (10 μg ml^−1^, 1 h treatment, Abcam), 3 washing steps (5 min each with 10% goat serum in PBS), treatment with a TRITC-conjugated secondary anti-mouse antibody (1 : 250 dilution, 1 h treatment, Millipore), and counterstaining with Hoechst 33342 (1 : 1000 dilution, 1 h treatment). After the immunostaining, cells were imaged through the confocal laser scanning microscope.

To quantify apoptotic responses following TMZ treatment, flow cytometry experiments were performed on 3D spheroids generated under non-adherent culture conditions. Briefly, 10 000 U-87 MG cells per well were seeded in a 96-well plate pre-treated with 1% agarose to prevent cell adhesion and promote spheroid formation. At 24 h of incubation, spheroids were treated with 30 μM TMZ for 48 h. Control spheroids were maintained in standard culture medium without drug exposure. Following treatment, spheroids were dissociated into single-cell suspensions. Specifically, spheroids were harvested, rinsed twice with PBS, and incubated with trypsin for 10 min at 37 °C. Gentle pipetting was applied to ensure complete dissociation, followed by centrifugation to collect the cells. The resulting cell pellet was resuspended in the respective binding buffer (1×, 100 μL) containing 5 μL annexin-V conjugated with fluorescein isothiocyanate (FITC, Abcam). Samples were incubated for 10 min at 37 °C in the dark to avoid photobleaching. A further centrifugation step was carried out to remove the staining solution, and cells were resuspended in PBS for fluorimetric analysis. Flow cytometric acquisition was performed using a Beckman Coulter CytoFLEX cytometer. FITC-labeled annexin-V was excited at 488 nm, and fluorescence emission was detected at 525 ± 40 nm. The percentage of annexin-V^+^ (apoptotic) cells was quantified using the CytoFLEX analysis software. The % of apoptotic cells in the TMZ-treated group was determined and normalized by subtracting the baseline apoptotic level observed in the untreated controls.

### 4.6 Scanning electron microscopy imaging

Cells were fixed by incubation in 4% PFA for 30 min, followed by two 5 min washing steps with Milli-Q water. Subsequently, the samples were further treated with 2% glutaraldehyde for 2 h at 4 °C and then washed with Milli-Q water for 5 min. The samples were progressively dehydrated through a graded ethanol series (0%, 25%, 50%, 75%, and twice at 100%). Finally, a 25 nm gold layer was applied to the samples *via* sputter coating at 60 nA for 25 s to prepare them for SEM imaging. Imaging was finally carried out by using a Helios NanoLab 600i FIB/SEM (FEI).

### 4.7 Statistical analysis

Statistical analysis was carried out using analysis of variance (ANOVA) followed by Tukey's HSD (honestly significant difference) *post hoc* test. Data are presented as the mean value ± standard deviation of three independent experiments. The significance was set at *p* < 0.05.

## Author contributions

Attilio Marino: investigation; methodology; validation; conceptualization; data curation; formal analysis; writing – original draft. Kamil Ziaja: investigation; methodology; data curation; formal analysis; writing – original draft. Marie Celine Lefevre: investigation; methodology; validation. Maria Cristina Ceccarelli: investigation; methodology; validation. Matteo Battaglini: methodology; conceptualization; writing – review & editing. Carlo Filippeschi: methodology; validation; conceptualization. Gianni Ciofani: conceptualization; resources; supervision; project administration; writing – review & editing.

## Conflicts of interest

The authors declare a patent filing related to some of the technologies presented in this article (Italian patent application IT102024000016873, 22/07/2024). The authors declare no other conflicts of interest.

## Supplementary Material

LC-025-D5LC00540J-s001

LC-025-D5LC00540J-s002

## Data Availability

The data that support the findings of this study are openly available in Zenodo^46^ at https://doi.org/10.5281/zenodo.14526102.

## References

[cit1] Czarnywojtek A., Borowska M., Dyrka K., Van Gool S., Sawicka-Gutaj N., Moskal J., Kościński J., Graczyk P., Hałas T., Lewandowska A. M., Czepczyński R., Ruchała M. (2023). Pharmacology.

[cit2] Grochans S., Cybulska A. M., Simińska D., Korbecki J., Kojder K., Chlubek D., Baranowska-Bosiacka I. (2022). Cancers.

[cit3] King J. L., Benhabbour S. R. (2021). Pharmaceutics.

[cit4] Huang R., Harmsen S., Samii J. M., Karabeber H., Pitter K. L., Holland E. C., Kircher M. F. (2016). Theranostics.

[cit5] Marino A., Camponovo A., Degl'Innocenti A., Bartolucci M., Tapeinos C., Martinelli C., De Pasquale D., Santoro F., Mollo V., Arai S., Suzuki M., Harada Y., Petretto A., Ciofani G. (2019). Nanoscale.

[cit6] Liu E. K., Sulman E. P., Wen P. Y., Kurz S. C. (2020). Curr. Neurol. Neurosci. Rep..

[cit7] Xiong Z., Raphael I., Olin M., Okada H., Li X., Kohanbash G. (2024). EBioMedicine.

[cit8] Rong L., Li N., Zhang Z. (2022). J. Exp. Clin. Cancer Res..

[cit9] Aparicio-Blanco J., Sanz-Arriazu L., Lorenzoni R., Blanco-Prieto M. J. (2020). Int. J. Pharm..

[cit10] Norouzi M., Yathindranath V., Thliveris J. A., Kopec B. M., Siahaan T. J., Miller D. W. (2020). Sci. Rep..

[cit11] Chen J., Hu S., Sun M., Shi J., Zhang H., Yu H., Yang Z. (2024). Eur. J. Pharm. Sci..

[cit12] Obrador E., Moreno-Murciano P., Oriol-Caballo M., López-Blanch R., Pineda B., Gutiérrez-Arroyo J. L., Loras A., Gonzalez-Bonet L. G., Martinez-Cadenas C., Estrela J. M., Marqués-Torrejón M. Á. (2024). Int. J. Mol. Sci..

[cit13] Sun D., Gao W., Hu H., Zhou S. (2022). Acta Pharm. Sin. B.

[cit14] Liu P., Griffiths S., Veljanoski D., Vaughn-Beaucaire P., Speirs V., Brüning-Richardson A. (2021). Expert Rev. Mol. Med..

[cit15] Haddad A. F., Young J. S., Amara D., Berger M. S., Raleigh D. R., Aghi M. K., Butowski N. A. (2021). Neuro-Oncol. Adv..

[cit16] Accomasso L., Cristallini C., Giachino C. (2018). Front. Pharmacol..

[cit17] Mobini S., Song Y. H., McCrary M. W., Schmidt C. E. (2019). Biomaterials.

[cit18] Wadman M. (2023). Science.

[cit19] Xie Z., Chen M., Lian J., Wang H., Ma J. (2023). Front. Oncol..

[cit20] Law A. M. K., Rodriguez de la Fuente L., Grundy T. J., Fang G., Valdes-Mora F., Gallego-Ortega D. (2021). Front. Oncol..

[cit21] Langhans S. A. (2018). Front. Pharmacol..

[cit22] Unger F. T., Witte I., David K. A. (2014). Cell. Mol. Life Sci..

[cit23] Santo V. E., Rebelo S. P., Estrada M. F., Alves P. M., Boghaert E., Brito C. (2017). Biotechnol. J..

[cit24] De León S. E., Pupovac A., McArthur S. L. (2020). Biotechnol. Bioeng..

[cit25] Marino A., Tricinci O., Battaglini M., Filippeschi C., Mattoli V., Sinibaldi E., Ciofani G. (2018). Small.

[cit26] Marino A., Battaglini M., Carmignani A., Pignatelli F., De Pasquale D., Tricinci O., Ciofani G. (2023). APL Bioeng..

[cit27] Xu Y., Xie X., Duan Y., Wang L., Cheng Z., Cheng J. (2016). Biosens. Bioelectron..

[cit28] Ortigoza-Diaz J., Scholten K., Larson C., Cobo A., Hudson T., Yoo J., Baldwin A., Hirshberg A. W., Meng E. (2018). Micromachines.

[cit29] Coelho B. J., Pinto J. V., Martins J., Rovisco A., Barquinha P., Fortunato E., Baptista P. V., Martins R., Igreja R. (2023). Polymers.

[cit30] Zhong J., Yang D., Zhou Y., Liang M., Ai Y. (2021). Analyst.

[cit31] Yang B., Wang C., Liang X., Li J., Li S., Wu J. J., Su T., Li J. (2023). Micromachines.

[cit32] Du X., Kong J., Liu Y., Xu Q., Wang K., Huang D., Wei Y., Chen W., Mao H. (2021). Micromachines.

[cit33] Marrella A., Varani G., Aiello M., Vaccari I., Vitale C., Mojzisek M., Degrassi C., Scaglione S. (2021). Altex.

[cit34] Pan Y., Jiang D., Gu C., Qiu Y., Wan H., Wang P. (2020). Microsyst. Nanoeng..

[cit35] Zou L., Wu C., Wang Q., Zhou J., Su K., Li H., Hu N., Wang P. (2015). Biosens. Bioelectron..

[cit36] Park S. Y., Hong H. J., Lee H. J. (2022). BioChip J..

[cit37] Lee S. Y., Ju M. K., Jeon H. M., Jeong E. K., Lee Y. J., Kim C. H., Park H. G., Han S. I., Kang H. S. (2018). Oxid. Med. Cell. Longevity.

[cit38] Benson K., Cramer S., Galla H.-J. (2013). Fluids Barriers CNS.

[cit39] Pradhan R., Mandal M., Mitra A., Das S. (2014). Sens. Actuators, B.

[cit40] Abasi S., Aggas J. R., Garayar-Leyva G. G., Walther B. K., Guiseppi-Elie A. (2022). ACS Meas. Sci. Au.

[cit41] Stupp R., Mason W. P., van den Bent M. J., Weller M., Fisher B., Taphoorn M. J. B., Belanger K., Brandes A. A., Marosi C., Bogdahn U., Curschmann J., Janzer R. C., Ludwin S. K., Gorlia T., Allgeier A., Lacombe D., Cairncross J. G., Eisenhauer E., Mirimanoff R. O. (2005). N. Engl. J. Med..

[cit42] Lee S. Y. (2016). Genes Dis..

[cit43] Edmondson R., Broglie J. J., Adcock A. F., Yang L. (2014). Assay Drug Dev. Technol..

[cit44] Curto V. F., Ferro M. P., Mariani F., Scavetta E., Owens R. M. (2018). Lab Chip.

[cit45] Mobini S., Song Y. H., McCrary M. W., Schmidt C. E. (2019). Biomaterials.

[cit46] Marino A. , Ziaja K., Lefevre M. C., Ceccarelli M. C., Battaglini M., Filippeschi C. and Ciofani G., Zenodo, 2025, 10.5281/zenodo.14526102 PMC12302074 40719015

