# Supplementary material for: High-throughput impedance monitoring in 3D tumor cultures: a multiplex, microfluidic-free platform for drug screening
Source: Lab Chip. 2025 Jul 17;25(22):5747–61. doi: 10.1039/d5lc00540j (PMC12302074; doi:10.1039/d5lc00540j)
Supplement: LC-025-D5LC00540J-s001 [file LC-025-D5LC00540J-s001.pdf]

Supplementary Information (SI) for Lab on a Chip.
This journal is © The Royal Society of Chemistry 2025

SUPPORTING INFORMATION

High-Throughput Impedance Monitoring in 3D Tumor Cultures: A Multiplex, Microfluidic-Free Platform for Drug Screening

Attilio Marino[1,,#], Kamil Ziaja[1,2,3,*,#], Marie Celine Lefevre[1], Maria Cristina Ceccarelli[1,2], Matteo Battaglini[1], Carlo Filippeschi[3], Gianni Ciofani[1,#]*

[1] Istituto Italiano di Tecnologia, Smart Bio-Interfaces, Viale Rinaldo Piaggio 34, 56025 Pontedera, Italy

[2] Scuola Superiore Sant'Anna, The Biorobotics Institute, Viale Rinaldo Piaggio 34, 56025 Pontedera, Italy

[3] University of Aveiro, Department of Chemistry, CICECO-Aveiro Institute of Materials, Rua de Calouste Gulbenkian 1, 3810-074 Aveiro, Portugal

[1] Istituto Italiano di Tecnologia, Bioinspired Soft Robotics, Via Morego 30, 16163 Genova, Italy

* Authors contributed equally to this work and shared first authorship

# Corresponding Authors: attilio.marino@iit.it; kamil.ziaja@iit.it; gianni.ciofani@iit.it.

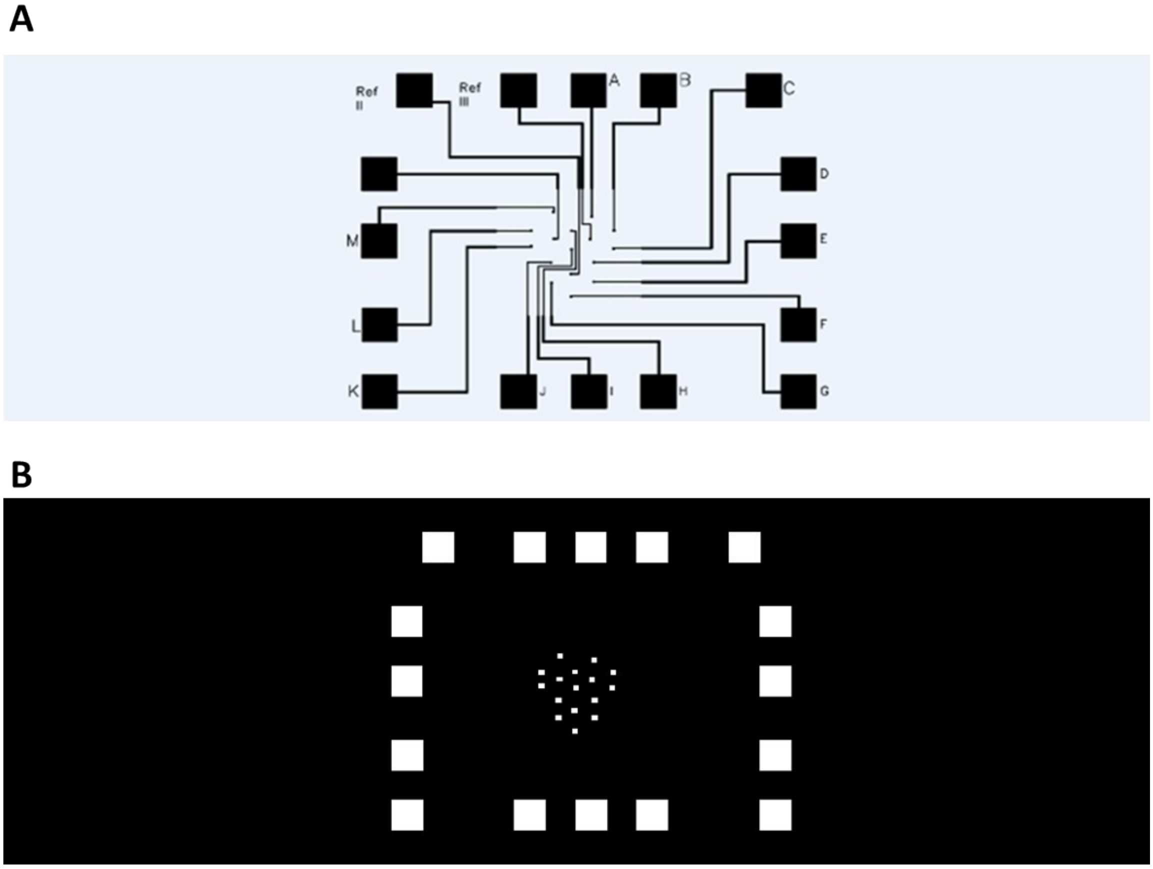

Figure S1. (A) Positive and (B) negative photomasks used for metal deposition and plasma etching, respectively.

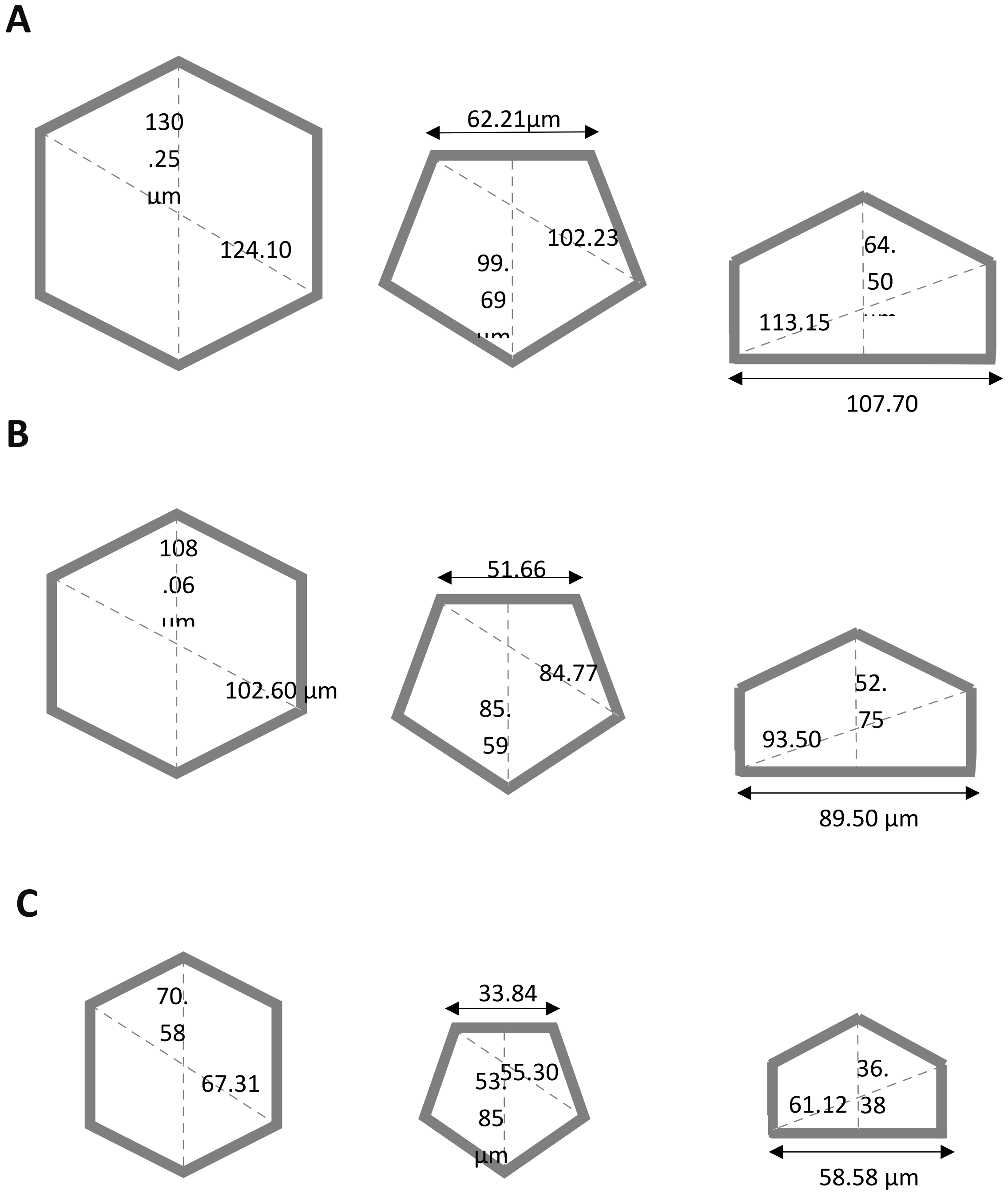

Figure S2. Pore sizes in the (A) outer, (B) middle, and (C) inner layers of the cupola-like scaffolds, used for promoting cell colonization of the measurement electrodes.

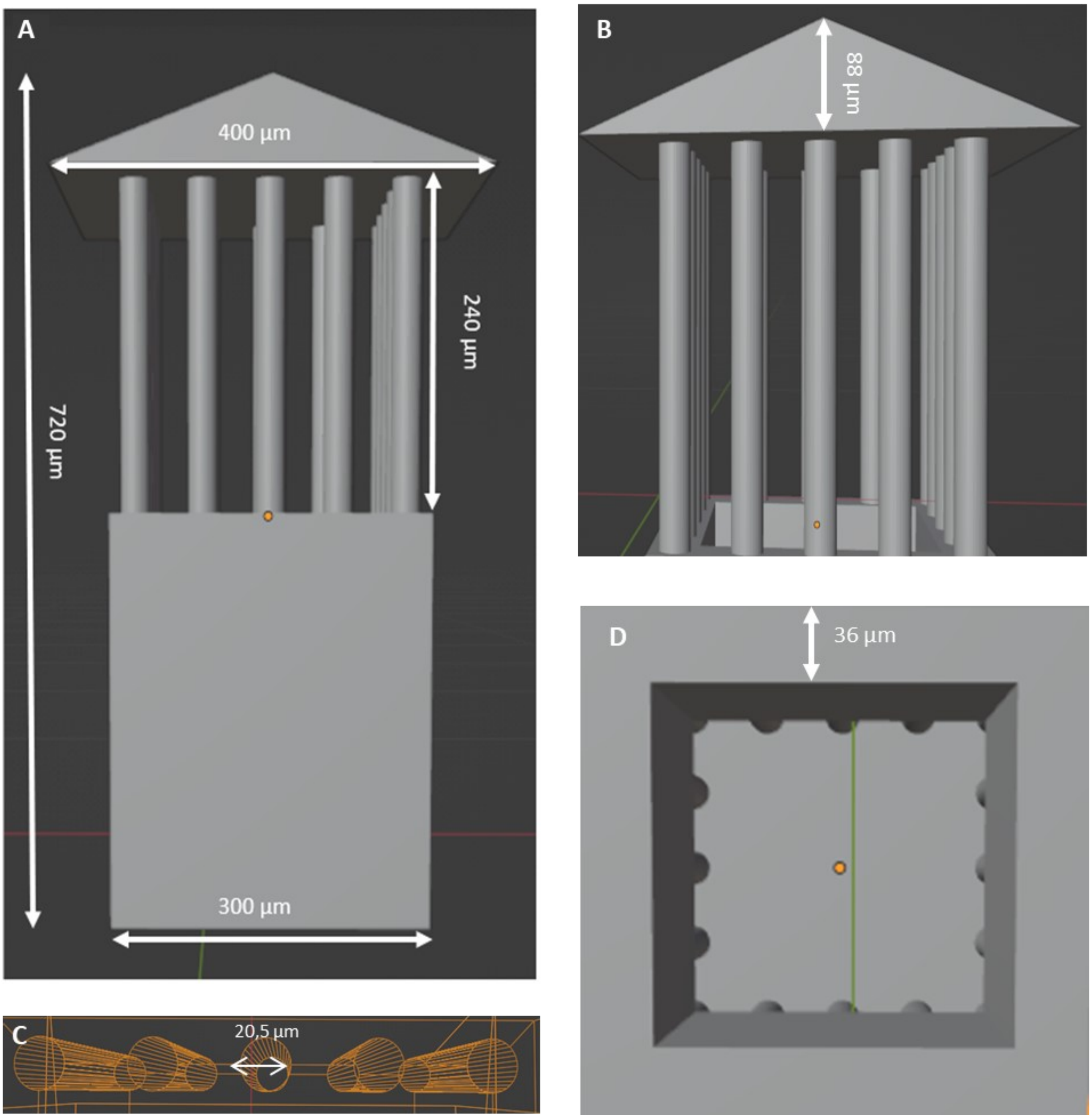

Figure S3. Design of the tower-like protective scaffold for the internal reference electrode. Size of the elements of the tower-like scaffold: (A, B) front view and (C) top view.

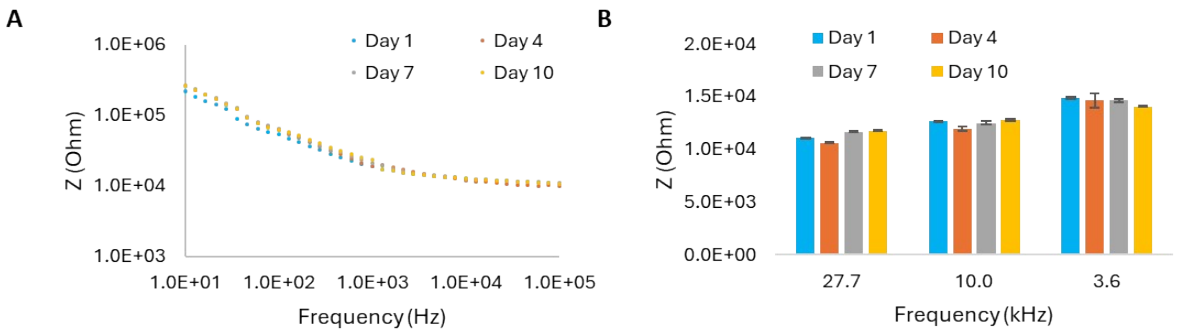

Figure S4. Long-term impedance stability of the integrated platform in cell culture medium. Impedance spectra (A) across the full frequency range and (B) for selected frequencies at four timepoints (1, 4, 7, and 10 days) upon continuous exposure to complete culture medium at 37 °C, showing no significant impedance drift.

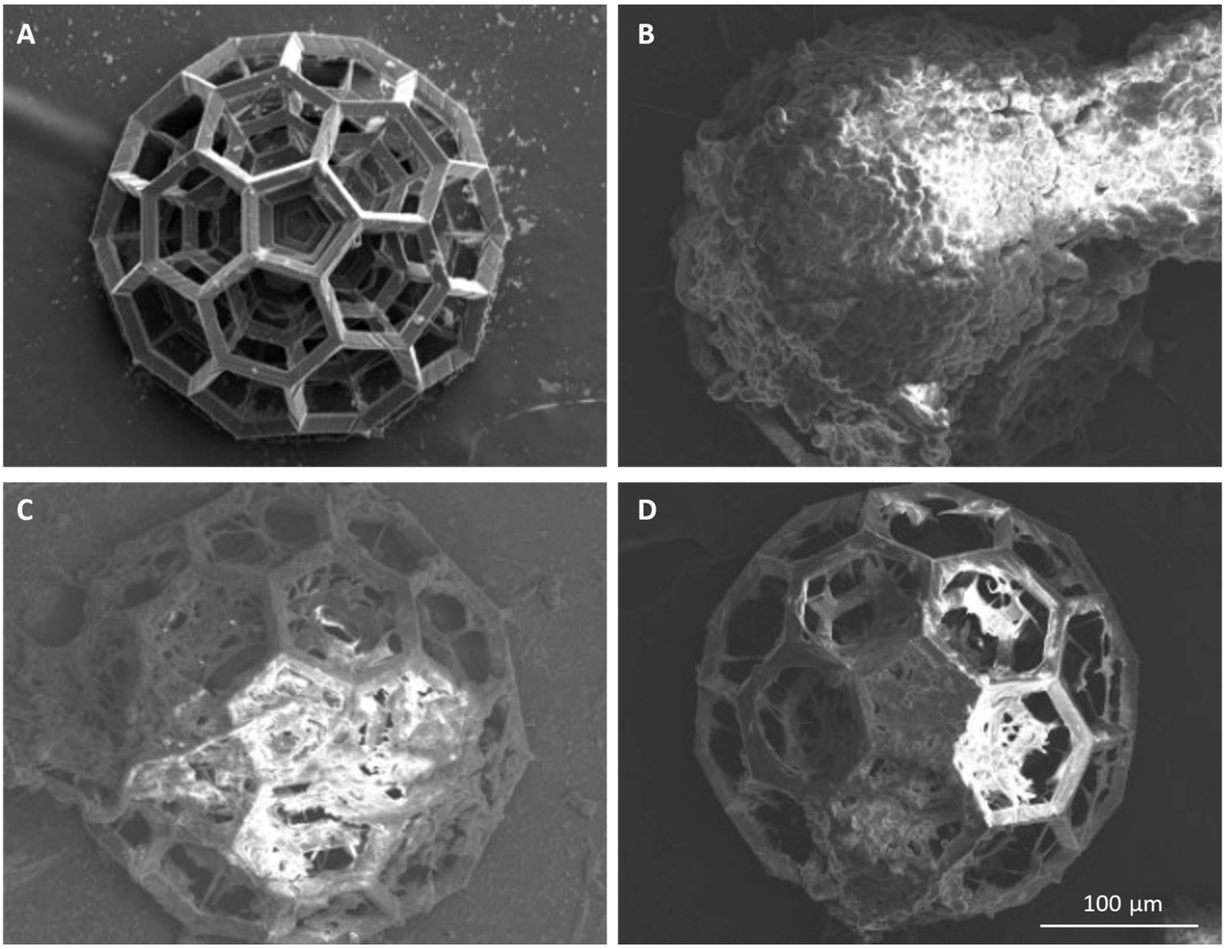

Figure S5. Representative SEM images of 3D cell cultures on the scaffolds. (A) 96-h U-87 MG cell culture; (B) culture after 3 h of nut-3a treatment; (C) culture after 24 h of nut-3a treatment. These images confirm the intimate cell-scaffold integration, the role of the pore design in promoting tissue formation, and the morphological changes induced by drug exposure

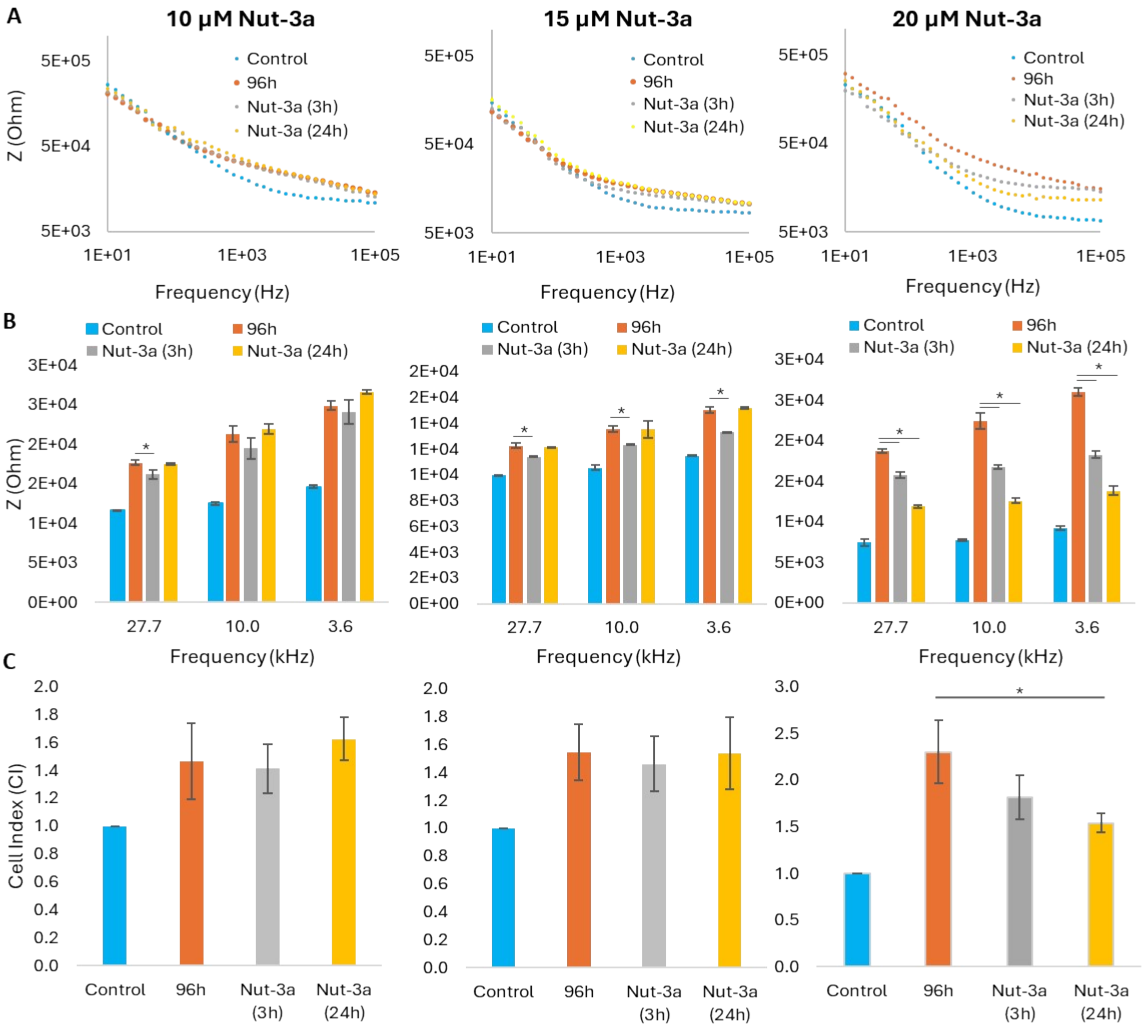

Figure S6. Impedance-based evaluation of dose-dependent response to nut-3a in U-87 MG 3D spheroids. (A) Impedance spectra across the full frequency range for 3D glioblastoma spheroids (96 h culture) following 3 and 24-h treatments with nut-3a at three concentrations (10, 15, and 20 μM); (B) corresponding impedance values at selected frequencies (3.6 kHz, 10 kHz, 27.7 kHz) showing time- and dose-dependent effects; (C) CI comparison across control and treated groups. * $p < 0.05$.

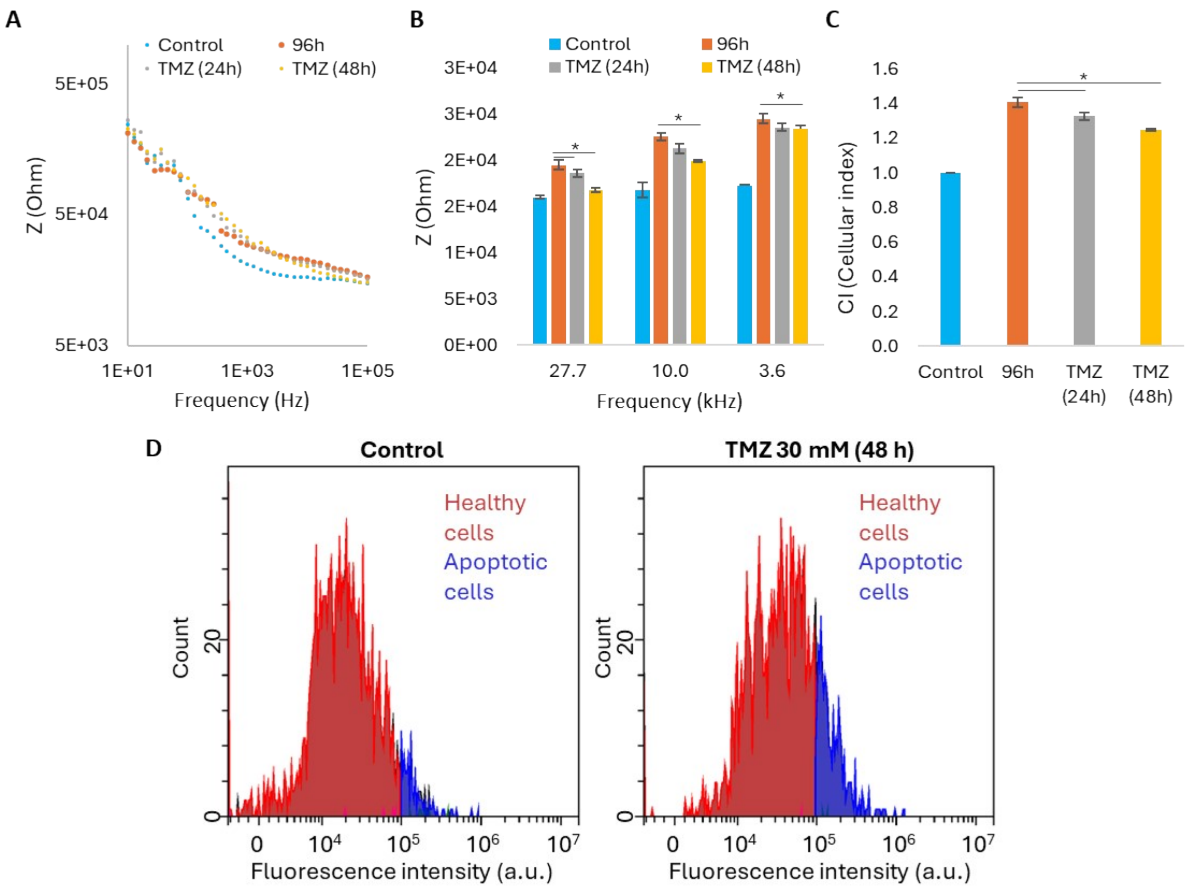

Figure S7. Impedance-based analysis of U-87 MG response to 30 mM TMZ. (A) Impedance spectra of 96 h glioblastoma spheroids treated with 30 mM TMZ, acquired at baseline, 24 and 48 h post-treatment; (B) impedance values at selected frequencies (3.6 kHz, 10 kHz, 27.7 kHz); (C) CI values; (D) healthy (red) and apoptotic (blue) populations detected by flow cytometry upon FITC-labeled annexin-V staining. * $p < 0.05$.

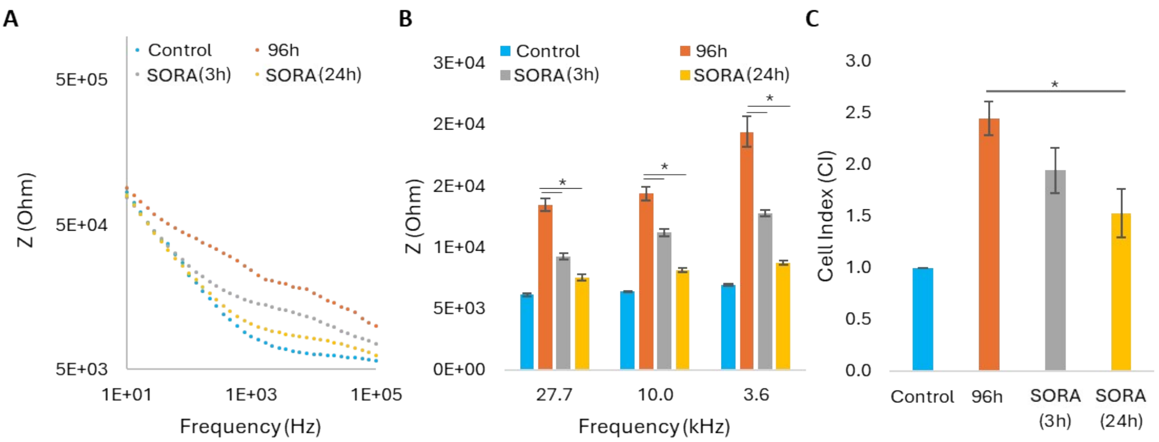

Figure S8. (A) Impedance spectra across the full frequency range following 3 and 24-h treatment with 15 µM sorafenib (SORA) in 96 h CaCo-2 spheroids; (B) corresponding impedance values at selected frequencies indicate a rapid and sustained reduction following treatment; (C) CI values. All these results support the ability of the platform to detect early drug responses in non-neural epithelial tumor models, confirming its versatility across cell types and treatment modalities. * $p < 0.05$.

Video S1. Automated fabrication of a scaffold on a measurement electrode. The video shows the slice-by-slice microfabrication process of the 3D scaffold using two-photon polymerization (2pp) directly around the platinum measurement electrode. The process includes laser scanning, layer-by-layer polymer crosslinking, and precise alignment with the underlying electrode geometry. This automated fabrication ensures high spatial resolution and reproducibility of the scaffold architecture
